# Trauma management incorporating focused assessment with computed tomography in trauma (FACTT) - potential effect on survival

**DOI:** 10.1186/1752-2897-4-4

**Published:** 2010-05-10

**Authors:** Karl-Georg Kanz, April O Paul, Rolf Lefering, Mike V Kay, Uwe Kreimeier, Ulrich Linsenmaier, Wolf Mutschler, Stefan Huber-Wagner

**Affiliations:** 1 Munich University Hospital, Department of Trauma Surgery - Campus Innenstadt, Ludwig-Maximilians-University, Munich, Germany; 2 Munich University Hospital, Department of Anaesthesiology - Campus Innenstadt, Ludwig-Maximilians-University, Munich, Germany; 3 Munich University Hospital, Department of Clinical Radiology - Campus Innenstadt, Ludwig-Maximilians-University, Munich, Germany; 4 Institute for Research in Operative Medicine, University Witten/Herdecke, Faculty of Medicine, Cologne, Germany

## Abstract

**Background:**

Immediate recognition of life-threatening conditions and injuries is the key to trauma management. To date, the impact of focused assessment with computed tomography in trauma (FACTT) has not been formally assessed. We aimed to find out whether the concept of using FACTT during primary trauma survey has a negative or positive effect on survival.

**Methods:**

In a retrospective, multicentre study, we compared our time management and probability of survival (Ps) in major trauma patients who received FACTT during trauma resuscitation with the trauma registry of the German Trauma Society (DGU). FACTT is defined as whole-body computed tomography (WBCT) during primary trauma survey. We determined the probability of survival according to the Trauma and Injury Severity Score (TRISS), the Revised Injury Severity Classification score (RISC) and the standardized mortality ratio (SMR).

**Results:**

We analysed 4.817 patients from the DGU database from 2002 until 2004, 160 (3.3%) were from our trauma centre at the Ludwig-Maximilians-University (LMU) and 4.657 (96.7%) from the DGU group. 73.2% were male with a mean age of 42.5 years, a mean ISS of 29.8. 96.2% had suffered from blunt trauma. Time from admission to FAST (focused assessment with sonography for trauma)(4.3 vs. 8.7 min), chest x-ray (8.1 vs. 16.0 min) and whole-body CT (20.7 vs. 36.6 min) was shorter at the LMU compared to the other trauma centres (p < 0.001). SMR calculated by TRISS was 0.74 (CI95% 0.40-1.08) for the LMU (p = 0.24) and 0.92 (CI95% 0.84-1.01) for the DGU group (p = 0.10). RISC methodology revealed a SMR of 0.69 (95%CI 0.47-0.92) for the LMU (p = 0.043) and 1.00 (95%CI 0.94-1.06) for the DGU group (p = 0.88).

**Conclusion:**

Trauma management incorporating FACTT enhances a rapid response to life-threatening problems and enables a comprehensive assessment of the severity of each relevant injury. Due to its speed and accuracy, FACTT during primary trauma survey supports rapid decision-making and may increase survival.

## Background

In central European trauma centres there is an increasing trend towards focused assessment with computed tomography in trauma (FACTT) [1,2]. According to the annual report 2008 of the German trauma registry, more than 44.9% reporting trauma centres utilize whole-body computed tomography (WBCT) in major trauma due to its speed and accuracy [3].

In 1997 Löw was the first to consider clinical use of WBCT during initial trauma resuscitation in Mainz, Germany [4]. The first subsequent patient series was conducted by Scherer in Munich, Bavaria in 1999 [5]. Leidner in Oskarshamn, Sweden [6], Ptak in Boston, U.S.A. [7], Klöppel in Leipzig, Germany [8] and Rieger in Innsbruck, Austria [9] all followed. Additionally Mutze from Berlin, Germany, introduced a detailed whole-body CT protocol for multiple trauma patients in a in a digitized radiology department [10]. Since then several approaches to the integration of WBCT into trauma room protocols have been presented [1,2,11-14]. Recently it could be demonstrated that integration of WBCT into early trauma care significantly increased the probability of survival in patients with polytrauma [15].

Implementing WBCT requires a trauma room (TR) that is suitably equipped governed by an algorithm that enables effective diagnosis and treatment of injuries. According to our ATLS^®^/ETC (Advanced Trauma Life Support^®^/European Trauma Course) based trauma workflow the MSCT (multi slice CT) scanner (situated in the emergency department adjacent to the trauma room) is utilized immediately after the management of respiratory problems (Airway, Breathing) to detect causes of bleeding (Circulation) or intracranial pathologies (Disability).

To date, the impact of focused assessment with computed tomography in trauma (FACTT) has not been formally assessed. We felt that this was necessary especially as we apply WBCT in haemodynamically stable as well as in haemodynamically unstable trauma patients. By using data from the trauma registry of the German Trauma Society (DGU), our study investigated whether our concept of using FACTT during primary trauma survey has a negative or positive effect on survival.

## Methods

**Figure 1:**
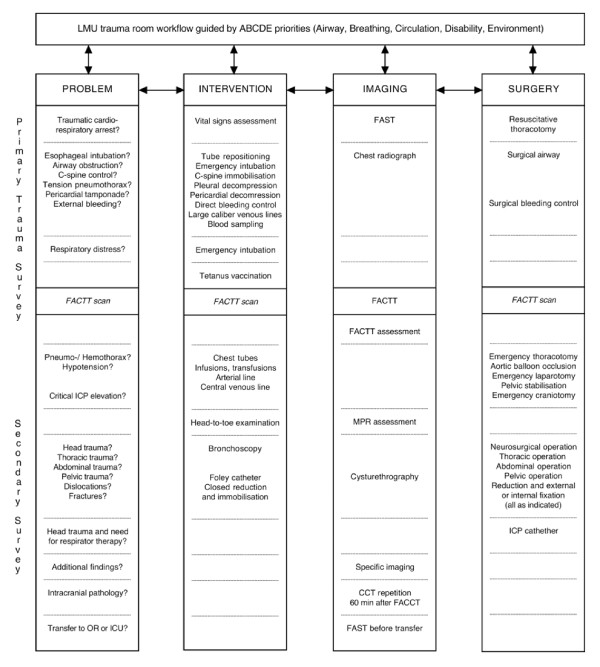
**Ludwig-Maximilians-University (LMU) trauma room workflow**. FACTT: focused assessment with computed tomography in trauma; ICP: intracranial pressure; OR: operation theatre; ICU: intensive care unit; FAST: focused assessment with sonography for trauma; MPR: multiplanar reformat/reconstruction; CCT: cranial computed tomography.

Our trauma workflow [fig. 1] enhances an established comprehensive trauma protocol [1] and amalgamates ATLS^®^/ETC standards. The algorithm is based on a scheme that is adjusted to priority and phase of trauma resuscitation. It incorporates diagnostics, evaluation and therapy and integrates the use of WBCT. According to the algorithm, respiratory (A, B), circulatory (C) and disability (D) problems need to be immediately identified and treated. Stethoscope (physical examination), sonography and chest x-ray serve as basic diagnostic tools. After controlling respiratory problems and obvious external bleedings, WBCT is performed in order to detect relevant internal bleeding in the chest, abdomen/pelvis or intracranial pathology. At our institution the attending trauma surgeon supported by the anaesthesiologist and radiologist decides whether FACTT is performed or not. Exclusion criteria are patients which immediately require life-saving surgery.

In the case of traumatic cardiorespiratory arrest (TCRA), the first task is to confirm the correct positioning of the endotracheal tube and upon suspicion of tension pneumothorax, immediate chest decompression. The use of focused assessment with sonography for trauma (FAST) facilitates a more informed decision when considering resuscitative thoracotomy or the termination of resuscitation efforts.

In non-TCRA cases, respiratory assessment still takes priority. If the patient has been intubated on-scene, an esophageal intubation must be excluded and in severe respiratory distress emergency intubation performed. In the case of a tension pneumothorax, immediate chest decompression is required. Simultaneously a chest x-ray is obtained and sonography performed.

Contractility of the myocardium, pericardial effusion, end-diastolic volume and free fluid in the abdomen is examined by FAST combined with cardiac arrest ultra-sound exam (C.A.U.S.E.) [16]. Life-threatening conditions such as cardiac tamponade or massive hypovolaemia are detected and treated at this stage. Relevant external haemorrhage is controlled by direct compression, tourniquet or if necessary by surgery. As an essential prerequisite for FACTT a large caliber intravenous line is necessary to administer contrast application.

Following transfer from the trauma room to the adjacent CT suite, the MSCT study was performed with a 4-row multi-slice computed tomograph (Somatom Volume Zoom, Siemens AG, Medical Solutions, Erlangen, Germany). WBCT is defined as a scan of the head, neck, thorax, abdomen and pelvis. The head is scanned with 4 × 1 mm collimation (2 mm of slice thickness reconstruction of the bone and 4 mm of the parenchyma). Thorax, abdomen and pelvis are taken with 4 × 2.5 mm collimation respectively and 5 mm slice thickness reconstruction of the parenchyma. Multiplanar reconstructions (MPR) of the cervical, thoracic and lumbar spine each with 3 mm slice sickness are compiled as a result. Detailed information on the LMU-WBCT-protocol are given in [17].

The first images on the CT console enable the trauma team to "look into the patient" and search for life-threatening problems and injuries that require emergency procedures and operations such as chest tube insertion, thoracotomy, laparotomy, pelvic C-clamp or CT-guided aortic balloon occlusion. After life-threatening conditions are managed or excluded, secondary survey supported by multiplanar reconstruction (MPR) is performed. Based on these findings, further management and treatment such as craniotomy or damage control surgery is determined.

We acquired our data set from the trauma registry of the German Trauma Society (DGU) which aggregates data of major trauma patients within German speaking countries and is a prospective, multicentric, standardized and anonymized data base. Every trauma patient admitted to one of the participating trauma hospitals*** with an injury severity score (ISS) ≥ 16 or ICU treatment is documented for the registry. Data anonymity is guaranteed both for the individual patient and the participating hospital. The registry comprises epidemiologic, physiologic, laboratory, diagnostic, operative, interventional and intensive care medical data as well as scoring and outcome data [18]. We used the dataset from 2002 to 2004 as the parameter "whole-body computed tomography" was first recorded in 2002. We analyzed 4.817 patients, comparing 160 patients from our institution at the Ludwig-Maximilians-University Munich (LMU) to 4.657 trauma patients from other trauma centres (DGU) participating in the registry. Inclusion criteria were patients presenting with an ISS ≥ 16, who had been admitted directly from the incident scene to the hospital and in which information about WBCT had been documented. This study has received the full approval of the ethics committee of the Ludwig-Maximilians-University of Munich, Germany.

The descriptive data analysis to compare our group against the other hospitals of the registry was performed using the Mann-Whitney-U test (both two sided) and χ^2 ^test. The outcome analysis was carried out by calculating the Trauma and Injury Severity Score (TRISS), the Revised Injury Severity Classification score (RISC) and the standardized mortality ratio (SMR, observed/expected mortality). Details on the TRISS and RISC score are given in [15,19-21] (TRISS) and [3,15,22] (RISC). Survival was defined as survival to discharge.

The standardized mortality ratio (SMR) is defined as a quotient of the observed to the expected mortality. If the SMR is 1, the calculated mortality rate by score is identical with the observed mortality rate. If the SMR is less than 1, more patients than expected survive; if the SMR is higher than 1, less patients than predicted survive. In epidemiology SMR serves as standard statistical tool.

We calculated 95% CIs when appropriate. Significance was assessed at p < 0·05. We did the statistical analysis using SPSS (version 15.0).

To validate our trauma room concept incorporating FACTT and to analyse whether WBCT during trauma resuscitation has a negative impact on survival we performed a risk adjusted approach and compared the SMR of our university hospital (LMU) with the SMR of the other trauma centres (DGU). Furthermore we investigated the difference between LMU and other DGU centres in respect to time between admission and FAST, chest x-ray and WBCT.

## Results

4.817 patients met the inclusion criteria, they comprised of 160 (3.3%) cases from our hospital and 4.657 (96.7%) from the other trauma centres. All of our patients were treated according to our trauma room workflow and in 138 (86.3%) WBCT was performed during primary trauma survey. The other 22 (13.7%) either were dead on arrival, died before WBCT could be performed or underwent emergency surgery without CT. Table 1 gives the main characteristics of the collective.

**Table 1:** Characteristics of 4.817 patients that met the inclusion criteria.

Group	LMU	DGU	p-value
**Number**	**160**	**4.657**	
**Characteristic**	mean ± SD or %	mean ± SD or %	
**Epidemiologic data**			
Age (years)	44.6 ± 18.3	42.5 ± 20.7	0.096
Male gender (%)	75.0	73.2	0.604
Blunt injury (%)	89.4	96.4	< 0.001
**Prehospital**			
Chest compression (%)	6.9	3.4	0.020
GCS (points)	10.9 ± 4.4	10.2 ± 4.8	0.099
Chest tube insertion(%)	10.0	7.7	0.293
Intubation (%)	51.3	59.6	0.035
Shock (%)	23.9	21.4	0.454
Infusion (ml)	1.504 ± 866	1.575 ± 1075	0.883
Prehospital time (min)	61. 9 ± 51.8	75.8 ± 46.5	< 0.001
Air lifted (%)	41.1	41.6	0.903
**In-hospital**			
Chest compressions (%)	8.9	5.1	0.036
Chest tube insertion TR (%)	33.5	24.5	0.010
Shock TR SBP ≤ 90 (%)	21.2	15.4	0.051
Base excess (mmol/L)	-5.8 ± 5.5	-3.7 ± 4.9	< 0.001
Infusion (mL)	4.950 ± 3844	2.668 ± 2424	< 0.001
Haemoglobin (mg/dL)	11.3 ± 2.9	11.3 ± 3.0	0.779
PRBC (%)	40.5	29.6	0.003
Number of PRBC	4.2 ± 9.2	2.6 ± 6.6	0.005
Thromboplastin time	68.9 ± 22.9	74.9 ± 23. 3	0.01
Emergency Operation (%)	10.6	6.2	0.025
Operation rate (%)	87.5	77.2	0.002
Operation per patient	5.2 ± 7.2	2.9 ± 3.5	< 0.001
ICU days	16.8 ± 23.6	12.3 ± 14.2	0.340
Respirator days	14.8 ± 22.9	8.4 ± 12.2	< 0.001
MOF (%)	77.7	25.0	<0.001
AIS head ≥ 3 (%)	56.3	59.2	0.459
AIS thorax ≥ 3 (%)	61.9	56.5	0.177
AIS abdomen ≥ 3(%)	25.6	23	0.442
AIS extremities ≥ 3 (%)	40.6	36.1	0.239
ISS (points)	32.5 ± 16.4	29.7 ± 13.0	0.296
NISS (points)	40.0 ± 17.8	35.6 ± 14.6	0.006
Hospital days	22.3 ± 27.9	25.9 ± 30.1	0.025
**Outcome**			
Mortality rate (24 h)	11.3	11.4	0.959
Mortality rate (overall)	18.8	22.0	0.324
**GOS **(%)			
5 Good recovery	36.6	35.7	<0.001
4 Moderate disability	22.2	24.2	<0.001
3 Severe disability	9.8	12.7	<0.001
2 Vegetative state	11.8	3.6	<0.001
1 Dead	19.6	23.8	<0.001

We analyzed 4.817 patients, comparing 160 patients from our university hospital (LMU) to 4.657 trauma patients from other trauma centres (DGU) participating in the registry. SPB Systolic blood pressure; GCS Glasgow Coma Scale; ISS Injury Severity Score; NISS New Injury Severity Score; ICU Intensive Care Unit; AIS Abbreviated Injury Scale; MOF Multi Organ Failure (defined as organ failure of two systems of >2 SOFA-score points of at least 2 days duration [43]; TR Trauma room; PRBC Packed Red Blood Cells. p: χ^2 ^test or Mann- Withney-U test (two sided). The overall mortality rate is not exactly equal to the rate of GOS "dead" due to the fact that GOS has not been documented for all patients.

The mean age of the whole collective (n = 4.817) was 42.5 years, 73.2% were male and 96.2% suffered from blunt trauma. The GCS on-scene was 10.2 points, 59.3% required endotracheal intubation, 21.4% of the patients were in shock and the mean accident to admission time was 75.3 minutes. The mean base excess on admission was -3.8 and haemoglobin concentration 11.2 mg/dL. 6.4% of the patients needed an emergency operation and 77.5% of the patients were operated during stay. The mean ISS was 29.8. The overall mortality rate was 21.9%.

The results of the differences between LMU and DGU regarding time and survival are documented in table 2.

**Table 2:** Differences between LMU and DGU trauma centres regarding time and survival.

Group	LMU	DGU
**Number**	**160**	**4.657**
**Characteristic**	n or % [95%CI] or mean ± SD	n or % [95%CI] or mean ± SD
***Diagnostics***		
***FAST***		
N	125/160	2.676/4.657
%	78.1	57.5
Time (min)	4.3 ± 3.3	8.7 ± 14.1
p-value^1^	<0.001	
***Chest x-ray***		
N	111/160	2.464/4.657
%	69.4	52.9
Time (min)	8.1 ± 4.0	16.0 ± 19.9
p-value^1^	<0.001	
***WBCT***		
N	138/160	1.223/4.657
%	86.3	26.3
Time (min)	20.7 ± 17.6	36.3 ± 28.3
p-value^1^	<0.001	
**Outcome**		
***TRISS***		
N	95/160	2.246/4.657
%	59.3 [95%CI 51.8-67.0]	48.2 [95%CI 46.8-49.7]
Observed mortality n	15/95	404/2246
Observed mortality rate %	15.8 [95%CI 8.5-23.1]	18.0 [95%CI 16.4-19.6]
Expected mortality rate by TRISS (%)	21.4	19.5
SMR	0.74 [95%CI 0.40-1.08]	0.92 [95%CI 0.84-1.01]
p-value^2^	0.24	0.10
***RISC***		
N	157 (160)	4.115/4.657
%	98.1 [95%CI 96.0-100.0]	88.4 [95%CI 87.4-89.3]
Observed mortality n	30/157	878/4115
Observed mortality rate %	19.1 [95%CI 13.0-25.3 ]	21.3 [95%CI 20.1-22.6]
Expected mortality rate by RISC %	27.6	21.4
SMR	0.69 [95%CI 0.47-0.92]	0.995 [95%CI 0.94-1.06]
p-value^2^	0.043	0.88

We analyzed 4.817 patients, comparing 160 patients from our university hospital (LMU) to 4.657 trauma patients from other trauma centres (DGU) participating in the registry. FAST Focused Assessment with Sonography in Trauma; WBCT whole-body Computed Tomography; TRISS Trauma Revise Injury Severity Score; RISC Revised Injury Severity Classification; SMR Standard Mortality Ratio. p: χ^2 ^test or Mann- Withney- U test (two sided); p^1 ^refers to the difference between LMU and the other hospitals; p^2 ^refers to the difference between expected and observed mortality.

Time from admission to FAST, chest x-ray and WBCT was significantly shorter compared to the other trauma centres (p < 0.001). Especially the span of time to WBCT, which was 16 minutes shorter at our institution. TRISS could be computed for 95 (59.4%) patients at the LMU and 2.246 (48.2%) patients meeting the inclusion criteria at the other hospitals. In contrast RISC methodology could be applied for 157 (98.1%) patients of the LMU collective and for 4.115 (88.4%) at the other trauma centres. In respect to the difference between expected and observed mortality, TRISS calculation showed neither in the LMU nor in the DGU collective a significant difference (LMU p = 0.24, DGU p = 0.10). RISC calculation demonstrated a significant difference with respect to expected to observed mortality for our collective (LMU p = 0.043, DGU p = 0.88) with a SMR of 0.69 resulting in an unexpected higher survival rate.

## Discussion

In a similar fashion to the acronym FAST "focused assessment with sonography in trauma" we would like to introduce the acronym FACTT "focused assessment with computed tomography in trauma" for whole-body computed tomography during primary trauma survey. We use the term "focused" not in an anatomical or topographical sense. However, the term emphasizes to focus first on the search for life-threatening injuries, second on the need for damage control surgery, and third on other injuries and surgery respectively. The prerequisite for applying FACTT is an accessible MSCT scanner, either situated in or close to the trauma room. Implementation requires a well organized interdisciplinary trauma team consisting of trauma surgeons, anaesthesiologists and radiologists.

The integration of computed tomography within clinical routine has revolutionized diagnostic radiology [23,24]. Since the invention of multi-slice computed tomography (MSCT), whole body computed tomography (WBCT) is possible as MSCT in comparison to single-slice CT is acquired faster. In 2001 Ptak showed that WBCT can be practiced safely in haemodynamically stable trauma patients [7]. This has been confirmed by many other studies [1,8,9,13,25-27]. Furthermore WBCT turned out to be a technique that is more time saving than applying conventional diagnostic comprising sonography, conventional radiography or non-multislice CT [1,6-9,26,27] Different trauma protocols using WBCT have been compared and showed that single pass acquisition protocols entailed lower radio exposure than segmented, partially overlapping examinations [28,29]. Nevertheless, the potential risk of cancer by radiation exposure should not be neglected. Therefore an adequate use of WBCT is necessary to justify its use for ensuring fast identification of the injury pattern. The potential gain in diagnostic safety should ideally result in a higher probability of survival. That this is possible could recently be proven [15].

Our results indicate that the use of FACTT according to our trauma protocol may increase survival. RISC methodology confirms that in our major trauma patients the observed mortality rate was significantly lower than the expected mortality rate (p = 0.043). On the one hand this suggests that use of FACTT combined with our trauma algorithm during primary trauma survey reduces mortality and on the other hand that our trauma room workflow leads to an efficient use of WBCT. The efficiency of our trauma protocol can be confirmed when considering the significantly shorter span of time needed to acquire sonography, chest x-ray and computed tomography (FAST: 4.3 vs. 8.7 min.; Chest x-ray: 8.1 vs. 16.0 min; WBCT: 20.7 versus 36.3 min.; p < 0.001) compared to the other trauma centers. Reasons for this might be the possibility to perform x-ray and sonography in the trauma room, the fact that sonography is done by the attending radiologist and not by the teamleader, the close location of the CT scanner to the trauma room and the clear aim to finish diagnostics as early as possible.

Additionally clinical parameters as the shock rate or the infusion amount as well as therapeutic measures (number of the operations) could possibly have had an influence on our results. Other potential explanations for the increased probability of survival might be the reduced time intervals from TR to ICU admission according to Ruchholtz [30]. However, these times have not been measured in our study.

We assume that trauma management incorporating FACTT provides a targeted approach during primary survey as well as during secondary survey and results in a better outcome for major trauma patients. The main advantages of FACTT are that a CT scan of all cavities enables a rapid response to life-threatening problems and that the extensive imaging enhances a comprehensive assessment of the severity of each relevant injury. Depending on the findings of FACTT, it is possible to accomplish a priority adjusted approach by deciding which injuries must be treated first. By estimating a preliminary ISS, the decision between damage control surgery and definitive care is also supported.

Furthermore FACTT often reveals unexpected or hidden diagnoses with a major therapeutic impact. Salim et al. showed that WBCT resulted in a 19% change of treatment of the investigated 1000 patients without obvious external signs of injury [31]. Deunk et al. found out that chest or abdominal CT scans resulted in a change of treatment up to 34% in blunt trauma patients [32]. Pfeifer and Pape in their review report on a rate of 15-22% of clinically significant missed injuries in polytrauma patients [33].

Realisation of such a time sensitive trauma workflow is challenging especially for the CT technicians. Siebers et al. stated that under "front line" conditions every fifth cranial CT (CCT) and every fourth trunk CT (TCT) study was completed with a median delay of 5 min. An independent process analysis revealed that unpreventable delays were due to system failure or patients who were unable to cooperate. Preventable delays were due to errors such as deviation from trauma room algorithm or intravenous lines that were too short [34].

It is not only the CT technicians who are challenged, all other team members need to be well managed and organized. Discroll et al. published a prospective analysis of 207 trauma patients showing that trauma teams in which staff simultaneously carry out allocated tasks have the quickest resuscitation times [35]. The recently introduced European Trauma Course (ETC) addresses this issue and focuses especially on the team approach. The team oriented ETC is evidence based, practical and flexible enough to meet regional European needs [36].

Beside a well organized trauma team, it is essential that the trauma room workflow is adapted to the specific structure of the hospital. Several studies in different settings demonstrated that implementation of a trauma room algorithm incorporating WBCT catalyses management to develop a faster, more accurate and efficient management procedure for major trauma patients to maximise the value of the WBCT [11,12,14].

Despite the advantages of FACTT, exposure to radiation remains a critical aspect and needs to be considered. For a WBCT an effective radiation dose of 10-20 mSv can be assumed, while 2 mSv for a conventional radiography series (consisting of chest, spine, pelvis) and 5-16 mSv for a selective organ CT are reported [11,26,37,38]. The effective dose on particular organs can accumulate and therefore potentially increase individual cancer risk [39]. The dose depends on patients size and moreover on scanning parameters and on the applied trauma protocol. Richards et al. investigated different protocols, comparing spiral CT to multislice CT in order to quantify the radiation exposure effect of the protocols on lifetime cancer risk. In their institution the multislice CT protocols exposed the patients to less radiation than single slice CT [40].

A study design that aims to investigate the effect of early assessment with WBCT in major trauma has recently been published. This trial is called "randomized study of early assessment by CT scanning in trauma patients" (REACT). The primary objective is to prove the beneficial effects of early trauma room CT scanning on trauma patients by comparing the effects of a strategy involving early trauma room CT scanning with a standard diagnostic imaging strategy on patient outcome. This is done by the analysis of the days spent outside the hospital in the first year after the trauma. In the latter strategy, the WBCT scanner is not located in the trauma room, but elsewhere in the hospital. The secondary objectives are to document the impact of introducing trauma room WBCT-scanning on health outcome, logistics, capacity utilization, waiting times, economies of scale, substitution patterns, and investments. Furthermore the radiation dosage is calculated in both strategies based on the actual number and type of radiological examinations and related to the initial trauma performed in each patient during the first year [41].

An appropriate indication for the use of WBCT during trauma resuscitation remains controversial [42]. There have been attempts to implement either a triage rule [14] or certain parameters [11] as decision assistance. At our institution the attending trauma surgeon supported by the anaesthesiologist and radiologist decides whether FACTT is performed or not. The use of FACTT seems justified in our collective due to the fact that our patients have been severely injured with a mean ISS of 32.5. However further investigations on comprehensive indications for FACTT should be initiated.

There are several limitations to our retrospective study. TRISS calculation could be performed only in 59.4% of the Munich patients and in 48.2% of the other participating trauma centres, whereas RISC methodology was available in 98.1% of the LMU group and 88.4% of the DGU group. Thus, the data might by biased as TRISS could not be calculated for the majority of the trauma cases. But this also indicates that RISC is much easier to maintain than TRISS, which might be due to the fact that RISC does not compute the respiratory rate on-scene. The DGU trauma registry does not record the location of the MSCT scanners in respect to the trauma room or the structure of the trauma team and the management protocol. Furthermore, the extent to which other trauma centres have implemented the principles of ATLS^®^/ETC has not been reported.

## Conclusion

Trauma management incorporating FACTT enables a rapid response to life-threatening problems and enhances a comprehensive assessment of the severity of each relevant injury. Furthermore FACTT might be able to reveal unexpected or hidden diagnoses with a major therapeutic impact. Implementing FACTT requires a well organized trauma team and trauma workflow adapted to the local environment. Despite the limitations of our study the data demonstrates that our trauma room workflow enables an efficient management and that the well integrated FACTT during primary trauma survey does not harm the patient, but in fact may increase survival in major trauma.

## Competing interests

The authors declare that they have no competing interests.

## Authors' contributions

KGK and SHW participated in the idea, planning, data analysis and interpretation, statistical analysis, and writing the report. AOP participated in the data analysis and interpretation and writing the report. RL participated in the planning, statistical analysis and interpretation. MVK participated in data interpretation and writing the report. UK and UL participated in data interpretation and writing the report. WM participated in data analysis and interpretation and writing the report. All authors have seen and approved the final version of the manuscript.
